# Are women always better able to recognize faces? The unveiling role of exposure time

**DOI:** 10.1371/journal.pone.0257741

**Published:** 2021-10-28

**Authors:** Torben Hansen, Judith Zaichkowsky, Ad de Jong

**Affiliations:** 1 Department of Marketing, Copenhagen Business School, Frederiksberg, Denmark; 2 Beedie School of Business, Simon Fraser University, Vancouver, Canada; Iowa State University, UNITED STATES

## Abstract

A longer exposure time generally improves individuals’ ability to recognize faces. The current research investigates whether this effect varies between genders and whether it is influenced by the gender of the exposed faces. Based on a set of four experimental studies, we advance our knowledge of face recognition, gender, gender distribution of exposed faces, and exposure time in three main ways. First, the results reveal that women are more likely than men to suffer from a decrease in face recognition ability due to a lower exposure time. Second, the findings show that when exposure time is short (vs. long) women recognize a larger proportion of same gender faces and also recognize a larger proportion of same gender faces as compared with the proportion of same gender faces recognized by men. Third, findings reveal that when individuals are only exposed to same gender faces, women recognize more faces than men regardless whether exposure time is short, or long. In short, the findings of this research suggest that insight into the interplay between gender and exposure time length is critical to appropriately determine human beings’ ability to recognize faces.

## Introduction

The ability to recognize strangers from previous interactions is an important application to security, service roles, and various business and social scenarios. While prior research suggests that women are better than men at recognizing faces [1, 2], one intriguing research topic is the interplay among gender, face recognition, and exposure time. Say, both Tom and Janet have been exposed to the same number of faces at the same events. Critical questions are then: whether they have the same ability to recognize faces afterwards; whether they are more capable of recognizing faces of their own gender; and whether their ability to recognize faces is influenced by exposure time. The extant literature on face recognition emphasizes that a longer exposure time generally improves individuals’ ability to recognize faces [3, 4]. In addition, several other studies on face recognition demonstrate superior recognition for an individual to recognize their own gender relative to the other [5, 6].

Faces contain a rich reservoir of information on factors such as gender, age, mood, social category, traits, and trustworthiness [5, 7, 8], and are arguably the most important social stimuli in interactions [9]. While face recognition is essential for many daily and social activities, it is also known that individuals vary considerably in their ability to recognize faces [10, 11]. Therefore, we believe that a more complete understanding of face recognition should consider both exposure time and gender. While exposure time and gender have been extensively studied as factors that have an influence on an individual’s ability to recognize faces, the interplay between these two factors has received less research attention.

In response to this, our study contributes to the existing literature by examining how the interaction of exposure time with gender and gender distribution of exposed faces affects an individual’s face recognition performance. It is relevant to take the exposure time factor into account as time often is a scarce resource having a huge impact on outcomes. For instance, the theory of selective attention contends that human beings have limited cognitive capacity and are selective in their attention [12, 13], which prevents them from attending to all issues that arise. This especially applies to settings, where time is limited and thus a scarce resource.

Based on face processing theory and gender theory, our study elaborates on this interplay and demonstrates that an investigation of face recognition should distinguish between the genders of the persons who are exposed to various faces versus the genders of these faces themselves. Four experimental studies, which manipulate exposure time and face images in several combinations, are conducted to test the hypotheses. Specifically, this study expands the present body of research in three ways. First, we show that the effect of exposure time is dependent on both gender and gender distribution of exposed faces. We find that women, in general, show a decrease in face recognition ability due to a lower exposure time. However, we find that when exposure time is short (vs. long) women recognize a larger proportion of same gender faces and recognize a larger proportion of same gender faces as compared with the proportion of same gender faces recognized by men. Third, when individuals are only exposed to same gender faces, women recognize more faces than men irrespective of exposure time.

These results are of interest to those who rely on confident identification of previous facial exposures in their day-to-day interactions. Whether it be a safety concern, social, or business interaction, the correct identification of previous encounters can be beneficial in deciding which gender to place in certain positions of initial encounters. Gender differences in face recognition and its interaction with exposure time yields new insights and implies that managers should use a differentiated approach in guiding male versus female employees to optimize outcomes. For instance, the results of the present study suggest that females are more likely than their male counterparts to suffer from a decrease in face recognition ability due to a lower exposure time.

### Gender differences and exposure time

A recent meta-analysis found that women are better than men at recognizing faces (Hedges’ g = .36), with the advantage seen primarily for female faces [2]. Several background explanations have been offered for women’s superior face recognition ability, such as greater self-reported social engagement [14], increased encoding specificity of faces [15], and superior detection of facial expression [16]. Research has also proposed that individuals may differ according to whether their general mode of processing information can be described as mainly experiential [17, 18] versus mainly detailed [19].

While the different modes normally engage in integrated interaction [20], some effort has been devoted to investigating whether gender differences may influence whether one mode of processing is more dominant than the other. Prior research [21–23] has found general gender-related differences in the strategies used to process information, such that women are more likely than men to show a detailed elaboration of information content, whereas men are more likely to show an overall information theme. One face recognition study [20] attributed superior face recognition in females to higher elaboration during face encoding. Other face recognition research [16] found (a) that women showed longer dwell time to the eyes compared to males and (b) a positive relationship between number of fixations to the eyes and accuracy of facial expression recognition. These findings are consistent with research based on social comparison theory suggesting that females are more likely to engage in time-consuming comparisons of facial characteristics from a young age [24].

While previous research has found that women are better than men at recognizing faces, no previous research known to us has investigated whether this ability is consistent across exposure time. Parkinson’s Law states that work expands to fill the time available, suggesting that as the time available to perform a task increases, so would the requirement for information to be potentially used in the task. This is in line with research suggesting that under time-pressure individuals tend to show greater selectivity when processing information [25]. In accordance with this result, one study found that a longer exposure time increased participants’ ability to discriminate between two inverted whole faces (i.e., a more demanding task compared to discriminating between upright whole faces) [3], indicating that time availability facilitates a more detailed processing of information.

Therefore, when we are given sufficient time, we should be more likely to process a face stimulus sequentially; local part by local part, which in turn is likely to increase our ability to individualize faces and to discriminate among them. This view is supported by recent research suggesting that misaligning facial composites, a technique widely accepted to inhibit holistic face processing, can significantly elevate face recognition [26] and is also in line with research suggesting that when individuals perceive faces some local parts may indeed be more diagnostic than others, such as the region of the eyes [27–29].

On the basis of the above discussion suggesting that (a) females are more likely than males to show a detailed elaboration of information content and (b) that a detailed elaboration is more time-consuming, we predict that a long exposure time is relatively more beneficial to females than to males. Hence, the following hypotheses are proposed:

H1a: Compared to females, males will recognize more faces when exposure time is short (vs. long).
H1b: Compared with males, females will recognize more faces when exposure time is long (vs. short).

### Own group bias and exposure times

Prior research suggests that an own group bias takes place in face recognition such that individuals are generally better at recognizing faces of their own age than older or younger faces [30, 31] and faces of their own race versus another race [6, 32]. Previous research also suggests that women are better at recognizing female over male faces [33–35]. However, less consistent results have been found for men.

While some studies suggest that men are better at recognizing male faces than female faces [35], other studies found that men were more able to recognize female faces versus male faces [1], and still other studies suggested that men recognized male and female faces equally well [33, 34]. We believe that these effects may be influenced by time-pressure, since individuals under time-pressure may be less likely to extend the scope of the task (e.g., accounting for social interaction implications) and more likely to focus on the task at hand (e.g., focusing their attentional resources on remembering faces) [36].

In that respect, the speed-accuracy tradeoff paradigm posits that categorization based on discriminability (e.g., females vs. males) is a key determinant in lowering processing time [37]. Furthermore past research indicates that when under time-pressure, and when confronted with discriminating stimuli, selective perception would appear and individuals may tend to focus on information that are more similar to their perceived self-identity [25, 38]. Based on this reasoning, we propose that exposure time will influence the presence of own group (i.e., own gender) bias in face recognition such that both females and males will show a higher propensity to recognize same gender faces versus faces of the opposite gender when exposure time is short (vs. long). Hence, we hypothesize as follows:

H2: Individuals will recognize a larger proportion of same gender faces when exposure time is short (vs. long).

A meta-analysis of own-gender bias in face recognition [2] found that women are better at recognizing female faces when respondents are exposed to a mix of female and male faces, but that women also outperform men when only male faces are shown. The authors suggest that women’s better performance in face recognition takes place because women may focus more attentional resources on remembering female faces when presented with a mix of male and female faces. When only male faces are shown they perform better than men because all attentional resources can then be devoted to male faces. While the meta-analysis [2] did not include variations in exposure time, we propose that regardless of exposure time (i.e., short vs. long), women will recognize a larger proportion of same gender faces compared with the proportion of same gender faces recognized by men.

Our reasoning is based on research suggesting that (a) women and men may encode information using different socially-constructed cognitive structures, which influence their perceptions and allocations of attentional resources [39, 40]; (b) while women may generally show a higher interest in other women (as compared with the interest of men in other men) [1] evidence also suggests that women are more attentive and responsive to cues with a perceived social relevance [40]. Hence, even though both men and women may devote more attentional resources to same gender faces when exposure time is short (vs. long) this type of resource allocation can be expected to be especially prevalent among women; and (c) when exposure time is long, substantial research suggests that women may have an advantage over men in recognition of female faces whereas no general, similar pattern has been detected for men [2]. To conclude, we propose that exposure time will influence own gender bias in face recognition as follows.

H3. Regardless of whether exposure time is short or long, females will recognize a larger proportion of same gender faces compared with the proportion of same gender faces recognized by males.

## Methodology and results

### Ethics statement

The authors declare that the data were collected in a manner consistent with ethical standards for the treatment of human subjects according to the principles expressed in the Declaration of Helsinki. This study was approved by the Copenhagen Business School Ethics Council. The respondents were asked to state their gender, but not to identify themselves in any other way, i.e., by name, or anything else. Hence, full anonymity was offered. Also, respondents were informed that it was not mandatory to participate. All respondents stated their informed consent orally before starting the study. The informed consent was witnessed by all other respondents and further documented by no respondents having any concerns about the study procedure. Afterwards, the completed forms were collected with no possibility of identifying non-participating respondents.

### Pretests

The face images used in the studies were obtained from the Face-Place Face Database Project [41]. We followed the procedure used by Suri and Monroe [42] and conducted two pretests in order to develop the manipulations of exposure time. The purpose of the first pretest was to determine the average time required to process 30 face images (15 being female images) when these images are presented together at a computer screen. A total of 58 graduate students participated in the first pretest. The participants were instructed to carefully look at the presented face images and to spend the time needed for them to be able to recognize the images afterwards. The time needed was computer registered and average reported time was 79.8 seconds. Consistent with our expectations that women (vs. men) are likely to engage in more time consuming face processing, the time needed was higher for females (*M* = 84.7, *SD* = 14.5) than men (*M* = 74.5, *SD* = 16.0), *t* = 2.56, *p* = .01. Based on the results of pretest 1, five short exposure time conditions (10 seconds, 20 seconds, 30 seconds, 40 seconds, and 50 seconds) and five long exposure time conditions (90 seconds, 100 seconds, 110 seconds, 120 seconds, and 130 seconds) were selected for further testing in pretest 2.

In pretest 2, participants were exposed to 30 faces (15 being female) with instructions the same as in pretest 1. Pretest 2 indicated the appropriateness of the time pressure conditions using the Suri and Monroe [42] subjective time pressure three-item scale. A sample item for this scale is ‘How would you characterize the time available for completing this task?’ (1 = more than adequate time available; 7 = not adequate time available). Ninety-one graduate students were distributed relatively equally across the time pressure conditions. Two criteria guided the selection of the time pressure conditions. First, the high time pressure condition should preferably be at least one standard deviation (*SD* = 15.94) below the mean time participants needed to make their choice in pretest 1 [43, 44]. Second, the high time pressure condition should be associated with time pressure (i.e., both the mean and the median of the averaged perceived three-item scale should be above the scale midpoint), while not making it impossible for respondents to perform the decision task (i.e., all respondents should have completed their choice within the time limits).

The Cronbach’s alpha value of the three-item subjective time pressure scale was.85, indicating sufficient scale reliability [45]. Also, a principal component analysis of the three items suggested a one-factor solution (based on the eigenvalue > 1.0 criterion) with consistently high loadings (ranging from.74 to.80) suggesting that the three items are essentially unidimensional [45]. Hence, we averaged the subjective time pressure scale by averaging the items together as an estimate of the construct value. The results led to the selection of 45 seconds (low exposure time) and 120 seconds (long exposure time). 45 seconds was chosen since subjective time pressure was almost identical for the 40 seconds (*M* = 4.57) and 50 seconds (*M* = 4.53) conditions. Averaged scale means (i.e., ranging from 1 to 7) were *M* = 5.48 (low exposure time) and *M* = 3.32 (long exposure time), *t* = 9.60, *p* < .01. Notably, our pre-tests are in line with the recent study conducted by Palmer, Brewer, and Horry [11], which used five seconds per face for the purpose of studying face recognition ability, although this study did not include variations in exposure time. Our research is based on four experimental studies, which manipulate exposure time and face images in several combinations.

### Study 1

#### Method

Sixty-eight graduate students were recruited (34 female). A power analysis revealed that a medium effect of the interaction between gender and exposure time with a power of 0.80 would require a minimum sample of 68 respondents suggesting a reasonable sample size for study 1. The number of respondents in studies 2–4 (see below) was 131, 110, and 124, respectively; indicating sufficient sample sizes for these studies. Based on prior research [2, 3] partial *η*^2^ for the gender-exposure time interaction was set to 0.10 in the power analysis.

Respondents were exposed to two experimental tasks. In the first task, a total of 30 face images were shown for 45 s. (i.e., 1.5 s. per image on average). Respondents were instructed to study the face images intensively so that they would later be able to remember as many images as possible. After completing this initial requirement, respondents were shown 10 faces (five of these being new images) for 60 s. with instructions to indicate the five images they believe had already been displayed. The five already seen images were randomly chosen from the 30 images pool. All the 10 images (5 already seen; 5 new) were shown together using a screen projector. The respondents were handed out a form where they were instructed to note the recognized faces. Sixty seconds was chosen in the recognition task because our pilot test suggests that this is a suitable time span, long enough to allow recognition activities to take place but not so long that participants may lose mental focus. These considerations were confirmed by interviews with participants after they had completed the required experimental tasks.

In both the five repeated images and the five new images groups, respectively, three of the images were females with the gender distribution being unrevealed to the participants. In the second task, respondents were exposed to another 30 face images which were shown for 120 s. (i.e., four seconds per image on average). The remainder of the second task was identical to the first task. We selected an unequal gender distribution in the repeated and new images groups because we wanted to take into account the possibility that participants, when in doubt of the ‘correct’ answer(s), might display an unequal propensity to randomly choose between female or male face images. This could be the case since previous research suggests that women and men may show an unequal attraction to same gender faces and to faces of the opposite gender [1, 2]. For that purpose, study three (see below) repeats study one but with a counter-balanced gender distribution in both the repeated and new images groups such that two females and three males (as opposed to three females and two males in the current study one) were included in each group.

#### Results and discussion

**Fig 1:**
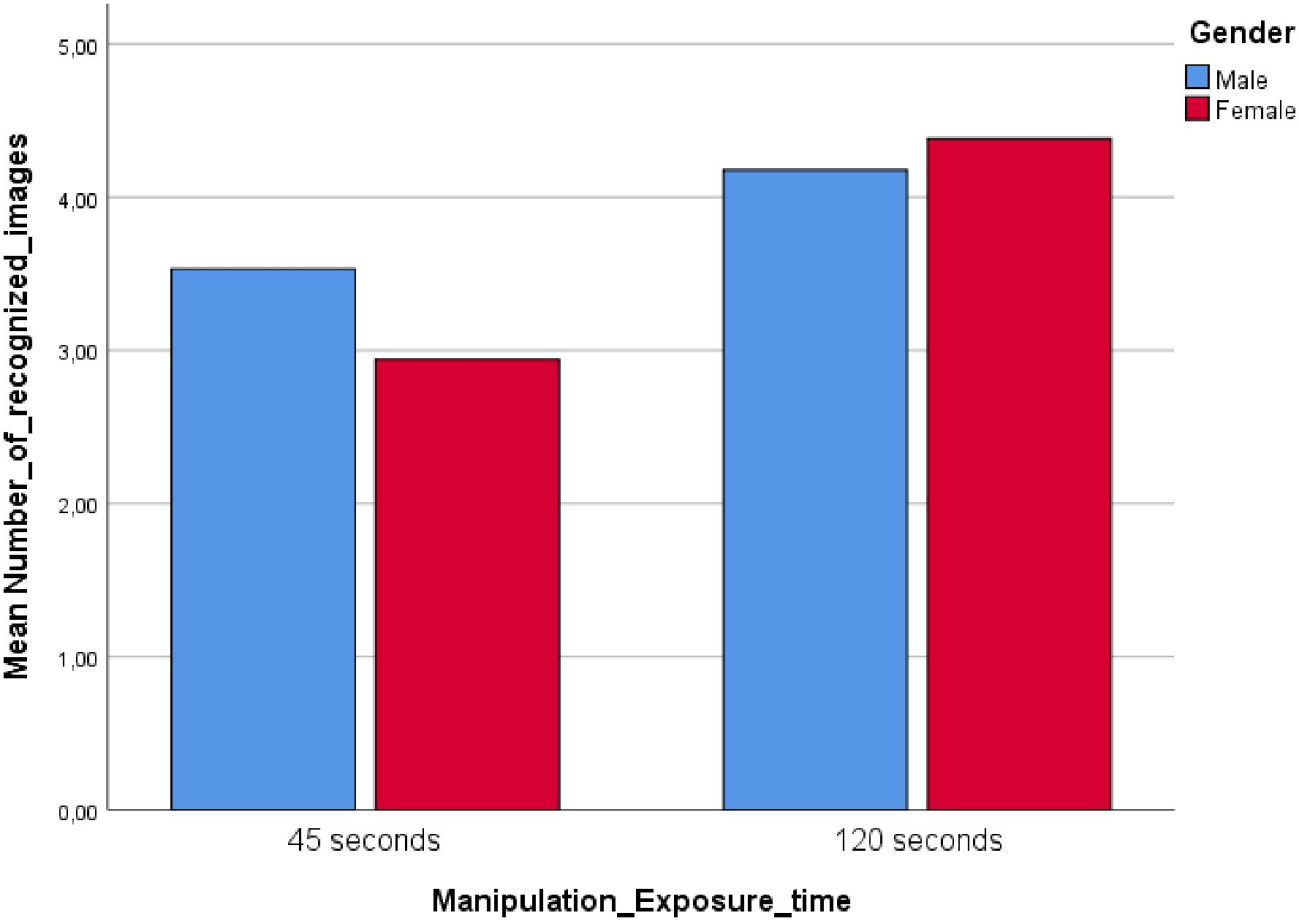
Study 1: Interaction between exposure time and gender.

*Hypotheses testing*. As expected from previous research, participants exposed to a long exposure time (LT) were able to remember more faces correctly (*M* = 4.28) than participants exposed to a short exposure time (ST), (*M* = 3.24), *F* = (1, 135) = 56.03, *p* < .01, *η*^2^ = .30. A two (short vs. long face exposure time) x two (males vs. females) ANOVA revealed a significant interaction between image exposure time and gender, *F* (1, 135) = 8.10, *p* < .01, *η*^2^ = .06 (Fig 1).

Supporting H1a, we found that males (*M* = 3.53) recognized more faces than females (*M* = 2.94) in the ST condition, *t* = 2.95, *p* = .01, whereas there were no face recognition differences between genders (*Mmales* = 4.17, *Mfemales* = 4.38) in the LT condition, *t* = 1.05, *p* = .30, although the mean difference was in the expected direction. Hence, H1b was not supported in Study 1.

Two additional 2 (short vs. long exposure time) x 2 (males vs. females) ANOVAs suggested a significant main effect of gender on the ability to recognize female faces (*Mmales* = 2.18, *Mfemales* = 2.71), *F* (1, 135) = 19.76, *p* < .01, *η*^2^ = .13, and on the ability to recognize male faces *(Mmales* = 1.69, *Mfemales* = .99), *F* (1, 135) = 41.91, *p* < .01, *η*^2^ = .24. Moreover, both female and male participants recognized a larger proportion of same gender faces in the ST condition (female: *M* = .83; male: *M* = .55) than in the LT condition (female: *M* = .72; male: *M* = .37); females: *t* = 2.40, *p* = .02; males: *t* = 4.26, *p* < .01. Hence, H2 was supported. Also supporting H3, females showed a higher tendency than males to recognize same gender faces in both the ST condition, *t* = 5.22, *p* < .01, and the LT condition, *t* = 10.63, *p* < .01.

Reflecting this parallel tendency across exposure time conditions, the interaction between image exposure time and gender did not influence participants’ propensity to show same gender recognition, *F* (1, 135) = 1.70, *p* = .20, *η*^2^ = .01. The results of study one support our expectations that males will recognize more faces than females in the ST condition and that both males and females will recognize a larger proportion of same gender faces in the ST (vs. LT) condition. We also found that females recognized more same gender faces than males in both the ST and LT conditions. However, contrary to our expectations, the results did not confirm that females will recognize more faces than males in the LT condition.

### Study 2

#### Method

Study 2 was identical to study 1 with the important modification of decreasing exposure time in the first task of the 30 face images for just 10 s. (i.e., approx..33 s. per image on average). The second task exposed participants to 30 face images for 120 s. as in study one (i.e., 4.0 s. per image on average). Specifically, we wanted to investigate whether (a) the decrease in exposure time from 45 s. to 10. s. led to a decrease in face recognition; (b) males still would be able to recognize more faces than females in the ST condition when processing time was reduced nearly to a practical minimum; and (c) whether the difference between females and males face recognition ability would be increased or decreased compared with study 1 results. The gender distribution in the repeated/new five images groups was similar to study 1 with three female face images included in each group. One hundred thirty-one graduate students (70 female) participated in Study 2. Exposure time was manipulated as between subjects.

#### Results and discussion

**Fig 2:**
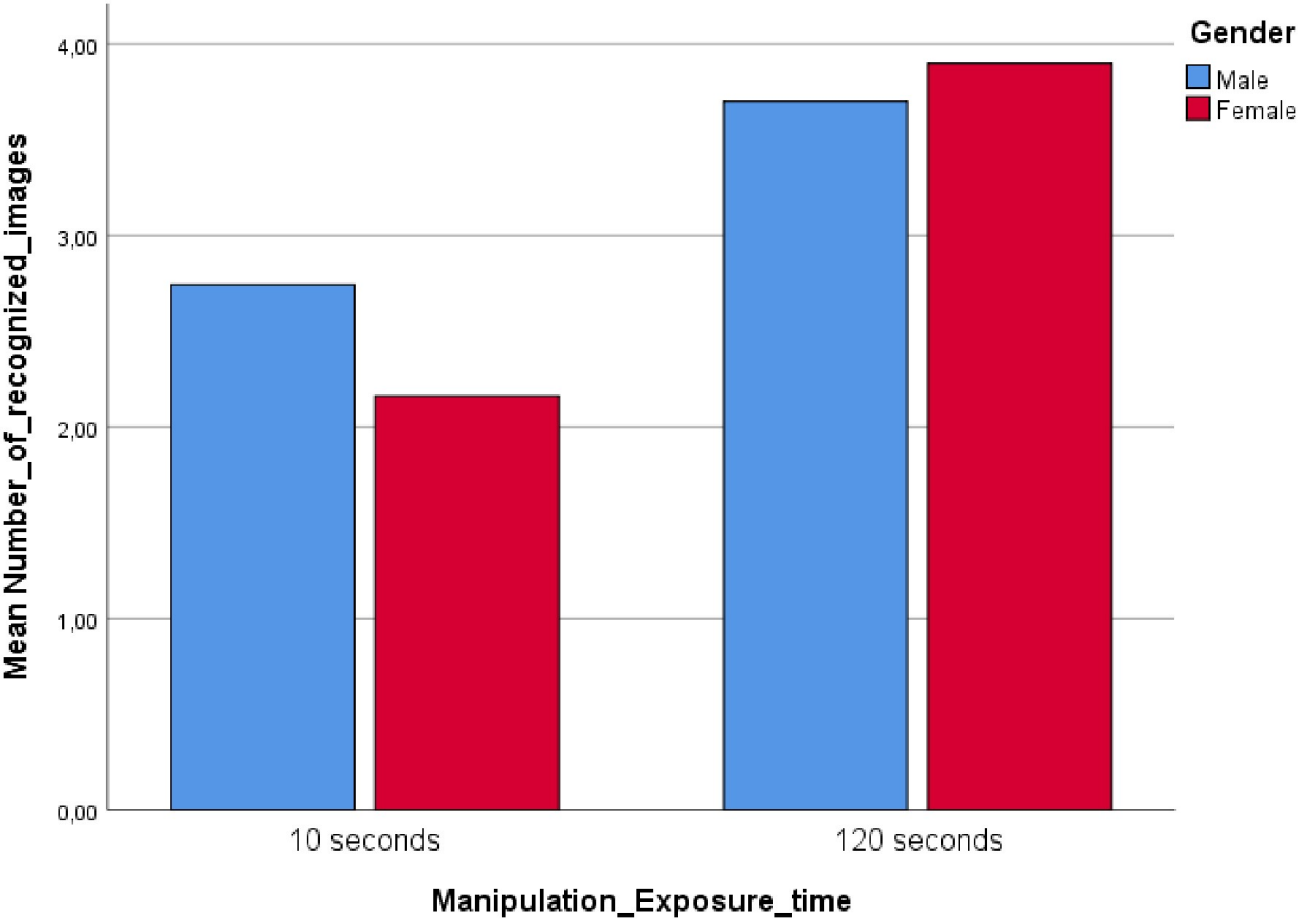
Study 2: Interaction between exposure time and gender.

Participants exposed to a long image exposure time (LT) were able to remember more faces correctly (*M* = 3.82) than participants exposed to a short image exposure time (ST), (*M* = 2.45), *F* = (1, 130) = 92.86, *p* < .01, *η*^2^ = .42. The results suggested a significant interaction between exposure time and gender, *F* (1, 130) = 7.98, *p* < .01, *η*^2^ = .06 (Fig 2).

**Fig 3:**
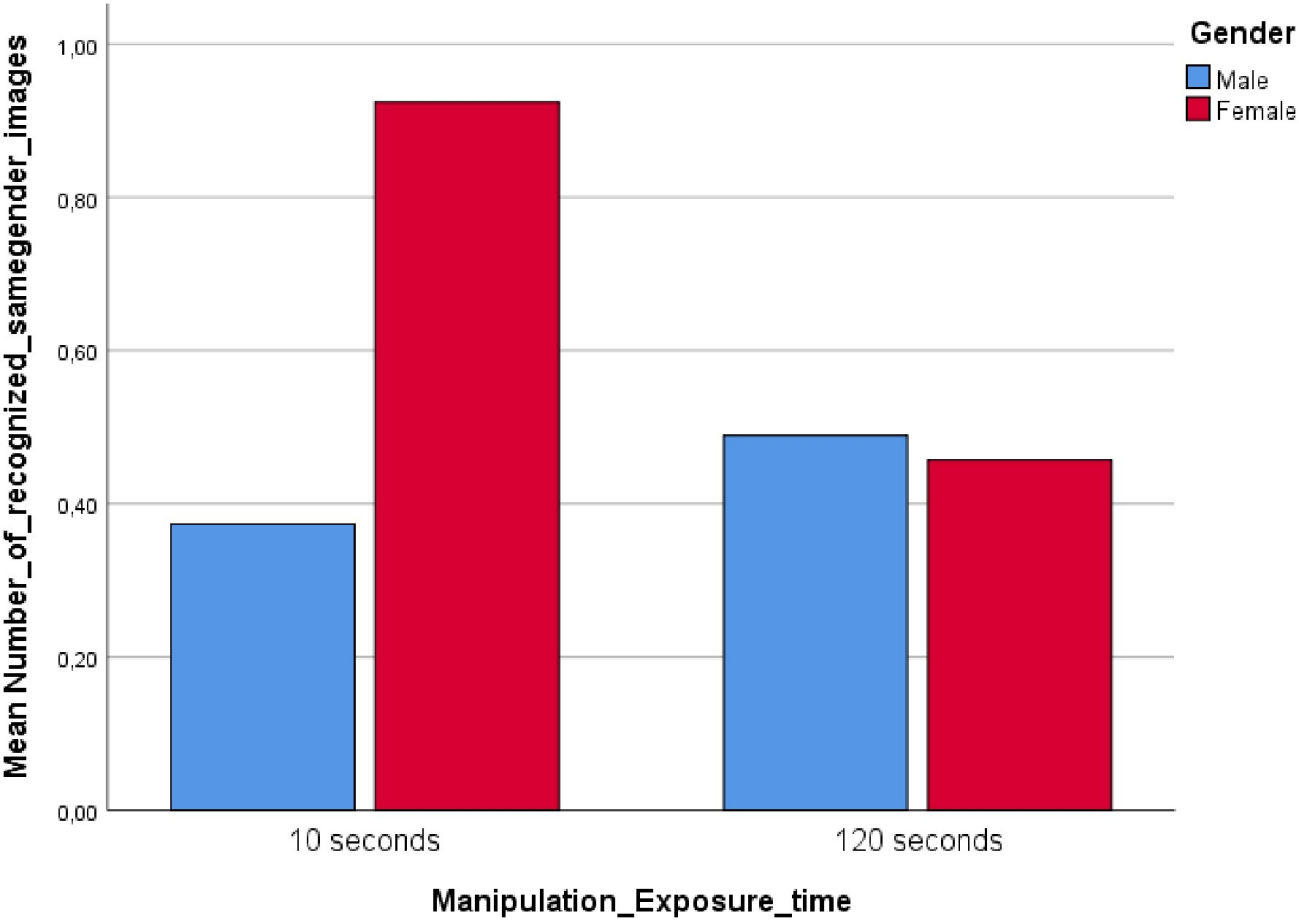
Study 2: Interaction between exposure time and gender.

The interaction between image exposure time and gender significantly influenced participants’ propensity to show same gender recognition, *F* (1, 130) = 17.84, *p* < .01, *η*^2^ = .12 (Fig 3).

Consistent with the results found in Study 1, and supporting H1a, males (*M* = 2.74) recognized more faces than females (*M* = 2.16) in the ST condition, *t* = 2.73, *p* < .01. H1b was not supported in study two as females did not recognize more faces than males in the LT condition (*Mfemales* = 3.90, *Mmales* = 3.70), *t* = 1.11, *p* = .27, although the mean difference was in the expected direction.

Two additional 2 (short vs. long exposure time) x 2 (men vs. women) ANOVAs suggested a significant main effect of gender on the ability to recognize female faces (*Mmales* = 2.11, *Mfemales* = 1.84), *F* (1, 130) = 3.91, *p* = .05, *η*^2^ = .03, but not on the ability to recognize male faces (*Mmales* = 1.39, *Mfemales* = 1.50), *F* (1, 130) = .09, *p* = .77, *η*^2^ < .01. Female participants recognized a larger proportion of same gender faces in the ST condition (*M* = .92) than in the LT condition (*M* = .46), *t* = 3.89, *p* < .01, whereas male participants recognized a smaller proportion of male faces in the ST condition (*M* = .37) than in the LT condition (*M* = .49), *t* = 2.14, *p* = .04. Hence, H2 was partially supported in study 2. H3 was also partially supported as females showed a higher tendency than males to recognize same gender faces in the ST condition (females: *M* = .92, males: *M* = .37; *t* = 4.03, *p* < .01), but not in the LT condition (females: *M* = .46, males: *M* = .49; *t* = .73, *p* = .47).

Consistent with our expectations, the decrease in exposure time from 45 s. (study 1) to 10 s. (study 2) in the ST condition led to a decrease in face recognition (45 s.: *M* = 3.24, 10 s.: *M* = 2.45; *t* = 5.11, *p* < .01). Across studies one and two, the interaction between gender and the variation in the ST condition (*F*(1, 129) = .01, *p* < .98, *η*^2^ < .01) and between gender and the variation in the LT condition *F*(1, 129) = .01, *p* < .97, *η*^2^ < .01) did not influence face recognition ability suggesting that the difference between female and male face recognition ability was robust both across variations in exposure time (with respect to the ST condition: 45 s. vs. 10 s.) and across studies (with respect to the LT condition: 120 s. in both studies 1 and 2).

Consistent with previous research [3, 4], the results of study 2 confirms that a decrease in exposure time (from 45 s. in study 1 to 10 s. in study 2) lead to a decrease in face recognition ability. We also found that men were still able to recognize more faces than women in the ST condition when processing time was reduced to a minimum. The results also suggest that the difference between women’s and men’s face recognition ability was neither increased nor decreased when compared with study 1.

### Study 3

#### Method

The purpose of study 3 was to replicate study 1 but with a counter-balanced gender distribution in the repeated/new five images groups such that two female face images (as opposed to three female face images in study 1) were included in each group. One hundred and ten graduate students (55 being female) participated in study 3.

#### Results and discussion

**Fig 4:**
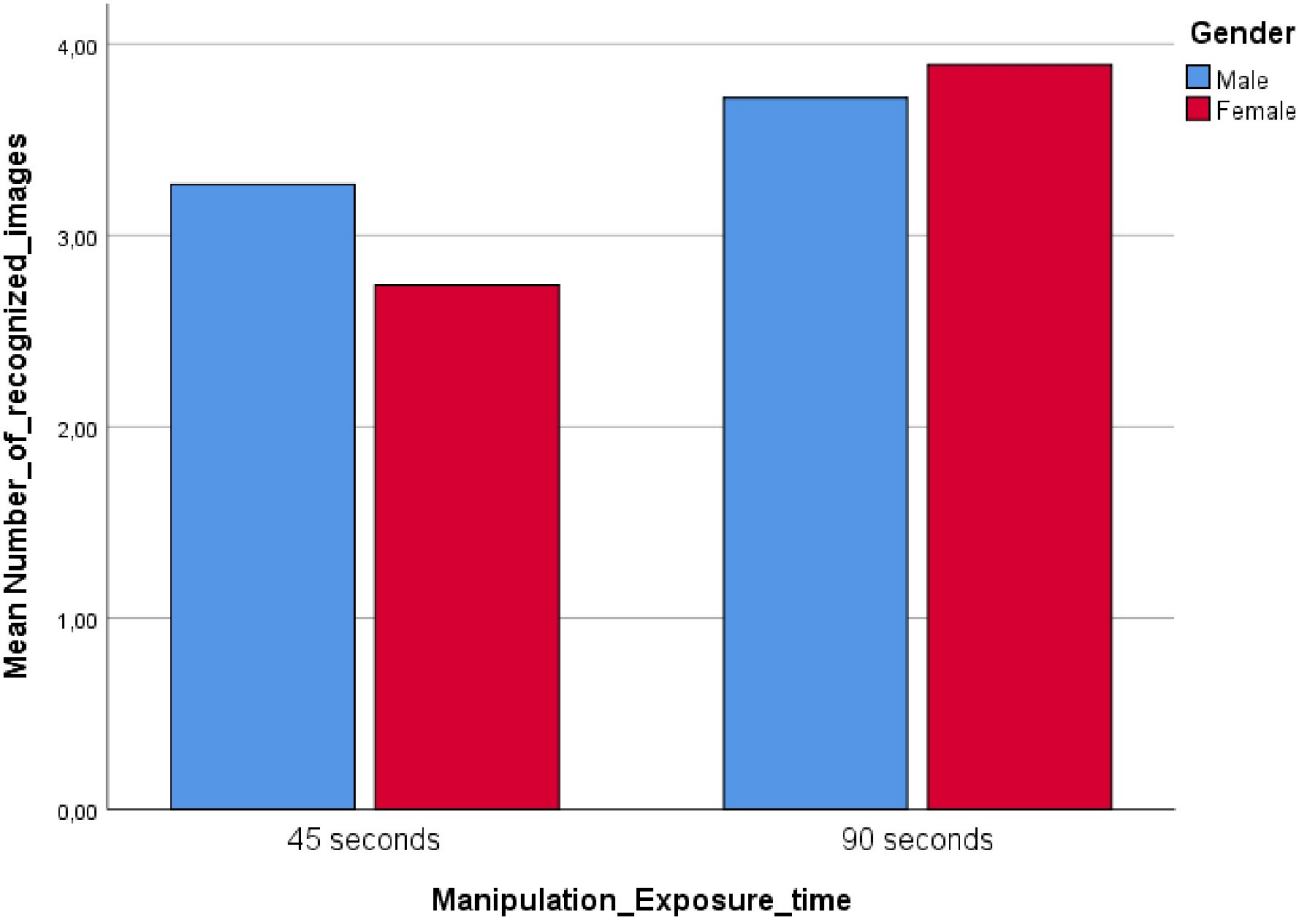
Study 3: Interaction between exposure time and gender.

The results suggested a significant interaction between image exposure time and gender, *F* (1, 109) = 4.18, *p* = .04, *η*^2^ = .04 (Fig 4).

Similar to study 1, participants exposed to a long image exposure time (LT) remembered more faces correctly (*M* = 3.81) than participants exposed to a shorter image exposure time (ST), (*M* = 3.02), *F* = (1, 109) = 20.98, *p* < .01, *η*^2^ = .16. Supporting H1a, men (*M* = 3.27) recognized more faces than women (*M* = 2.74) in the ST condition, *t* = 2.24, *p* = .03. H1b was not supported in Study 3 as there were no face recognition differences between genders (*M*male = 3.72, *M*female = 3.89) in the LT condition, *t* = .70, *p* = .49, although the mean difference was in the expected direction.

Two additional 2 (short vs. long exposure time) x 2 (male, vs. female) ANOVAs showed significant main effects of gender both on the ability to recognize female faces (*Mmale* = 1.15, *Mfemale* = 1.65), *F* (1, 109) = 13.89, *p* < .01, *η*^2^ = .12, and on the ability to recognize male faces (*Mmale* = 2.40, *Mfemale* = 1.76), *F* (1, 109) = 24.85, *p* < .01, *η*^2^ < .19. While female participants recognized a larger proportion (*t* = 2.81, *p* < .01) of same gender faces in the ST condition (*M* = .94) than in the LT condition (*M* = .71), the proportion of same gender faces recognized by men was not different (*t* = .82, *p* = .42) across conditions (ST condition: *M* = .82, LT condition: *M* = .77), although the mean difference was in the expected direction. Hence, H2 was partially supported. Women showed a higher tendency than men to recognize same gender faces in the ST condition, *t* = 2.09, *p* = .04, but not in the LT condition, *t* = .70, *p* = .43. Hence, H3 was also partially supported in study 3.

Supporting the robustness of our studies, the results of study 3 were similar to the study 1 results on the majority of the investigated aspects: (1) participants exposed to a long image exposure time remembered more faces correctly than participants exposed to a shorter image exposure time; (2) males recognized more faces than females in the ST condition, and (3) no gender difference in face recognition ability was found in the LT condition.

Moreover, and consistent with the study 1 results, female participants recognized a larger proportion of same gender faces in the ST condition than in the LT condition and women showed a higher tendency than men to recognize same gender faces in the ST condition. However, contrary to the study 1 results, the proportion of same gender faces recognized by men was not different across conditions (ST vs. LT) and we also did not find that women had a higher tendency than men to recognize same gender faces in the LT condition.

### Study 4

#### Method

Studies 1–3 produced mixed results regarding the ability to recognize same gender faces. While Study 1 suggested that females showed a higher tendency than males to recognize same gender faces in both the ST and the LT conditions, studies 2 and 3 indicated that this tendency was more likely to appear in the ST condition. Hence, we wanted to investigate more thoroughly whether females are more likely than males to recognize same gender faces across both ST and LT conditions. In Study 4, participants were only exposed to same gender face images (n = 30); with the repeated (n = 5) and new images (n = 5) groups consisting of same gender face images. One hundred twenty-four graduate students (58 female) participated in Study 4. As in studies 1 and 3, exposure times were 45 s. (ST condition) and 120 s. (LT condition) respectively.

#### Results and discussion

**Fig 5:**
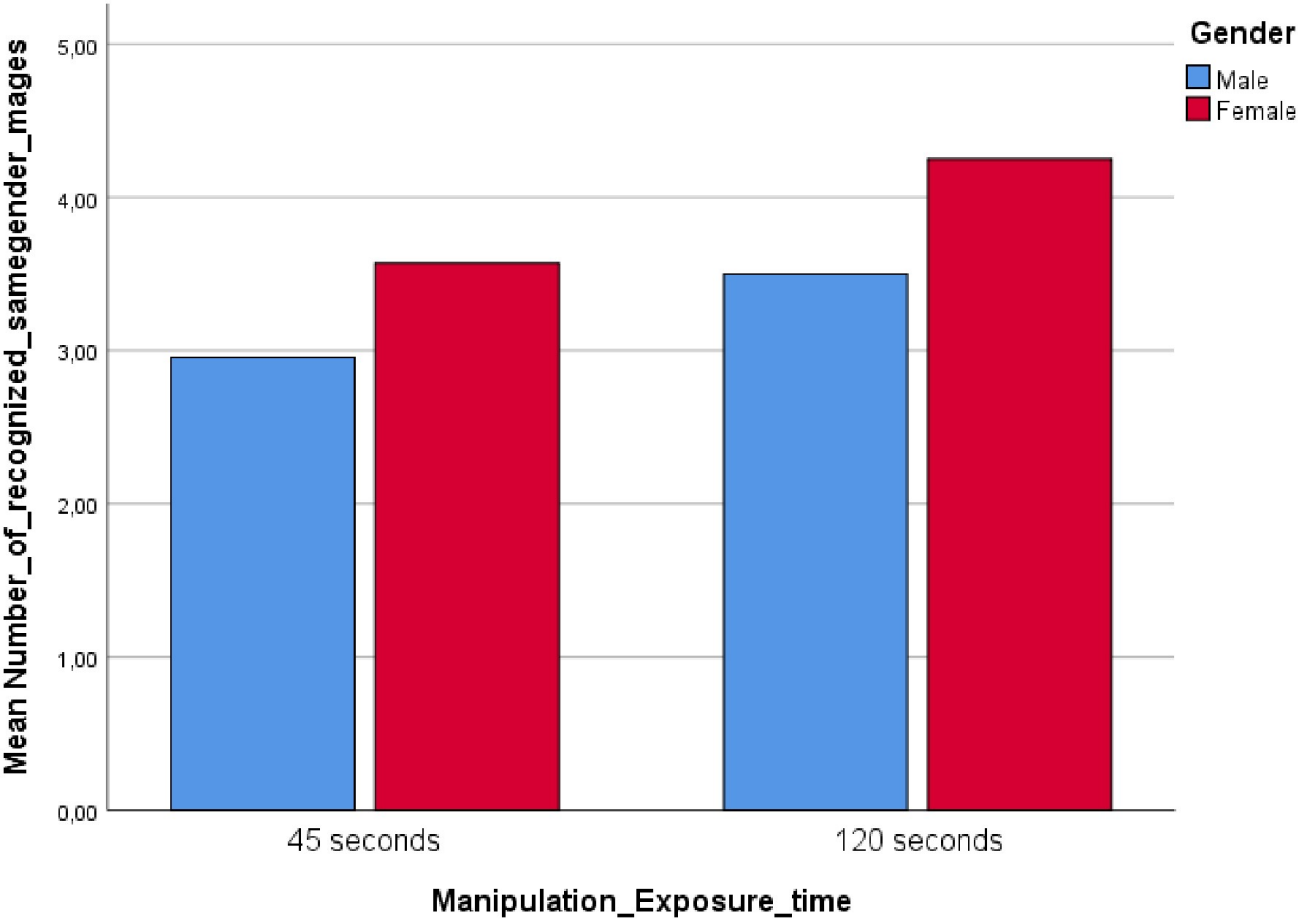
Study 4: Interaction between exposure time and gender.

Consistent with the results obtained in studies 1–3, participants exposed to a long image exposure time (LT) were able to remember more face images correctly (*M* = 3.83) than participants exposed to a short image exposure time (ST), (*M* = 3.25), *F* = (1, 123) = 4.54, *p* = .04, *η*^2^ = .04. Female participants recognized more face images in both the ST condition (women: *M* = 3.57, men: *M* = 2.96; *t* = 2.00, *p* = .05) and the LT condition (females: *M* = 4.25, males: *M* = 3.50; *t* = 2.01, *p* = .05). Reflecting this consistency across conditions we found a significant main effect of gender (*F* = (1, 123) = 6.42, *p* = .01, *η*^2^ = .05), whereas the interaction between image exposure time and gender was non-significant (*F* = (1, 123) = .06, *p* = .80, *η*^2^ < .01) (Fig 5).

Studies 1–3 all indicated that males recognized more faces than females in the ST conditions, whereas we found no gender difference in face recognition ability in the LT conditions. However, studies 1–3 all exposed participants to a mix of female and male face images as well as mixed gender distribution in both the repeated and new image groups. When participants were only exposed to same gender faces, females clearly recognized more faces than males in both the ST and the LT conditions.

## Discussion and conclusions

The present study reveals that assessing the interplay between gender, gender distribution of exposed faces, and exposure time is critical to get insight into women’s and men’s ability to recognize faces. We expand the extant body of research that revealed that women remember more faces than men by adding important nuances of both the length of exposure time and the gender distribution of exposed faces (see [2] for an overview). More specifically, in studies 1–3 we find that men recognize more faces than women when exposure time is short (vs. long). This result is consistent with our arguments that women tend to engage in a detailed elaboration of information content using more time-consuming face processing, which turns out to be more effective for longer exposure time. This finding also relates to social comparison theory, which suggests that males and females are likely to develop different sets of values and motivations, which may lead them to develop different perceptions and behaviors in similar contexts [46].

Our results clearly indicate that exposure time acts as an important contingency of the effectiveness of the human face processing. Shorter exposure times associate with higher face recognition performance of overall face processing, while for longer exposure times, detailed face processing yields higher face recognition performance rates. This proposition is consistent with the general contention that the impact of the individual specialized functions of the brain hemispheres on human performance is contingent on contextual factors, like exposure time [47]. While we attribute our findings to gender differences in face elaboration, we cannot rule out that other explanations may further detail the results. Given that no gender difference is observed in the LT condition, the results could also imply that men are more strategic in the ST condition. For example men (vs. women) may be more likely to focus on just a few face characteristics during exposure time and then seek to remember these few characteristics instead of the whole faces. Future research may wish to investigate such issues by using eye tracking techniques, which allows for an examination of respondents’ dwell time toward different face characteristic [16].

Contrary to our expectations, our results revealed that if exposed to *mixed* gender faces women do not significantly outperform men under long exposure time (vs. short) conditions. Albeit that women score, on average, higher than men do in all three studies (i.e., studies 1–3). In contrast, when only exposed to *same* gender faces, both in the repeated and in the new faces’ groups, women clearly outperform men regardless the length of the exposure time (study 4). This result substantiates previous face recognition research by adding nuance suggesting that women are more *same* gender focused and as such more experienced in perceiving and encoding same gender faces than men [2, 34].

In short, our findings clearly suggest that it is essential that face recognition research considers both the length of exposure time and gender distribution of exposed faces when studying the relationship between gender and face recognition performance. This enables us to acquire more insight into the effectiveness females versus males face recognition processing as being a function of exposure time. It substantiates insight into exposure time as an important contingency of the effectiveness of gender-specific face recognition processing.

In this study, the test images shown were the same identical images of those people that were shown at encoding. This is an important distinction as recognizing a previously-seen image is not the same as recognizing a previously-seen face or person. As argued by Burton [48], it is always easier to recognize a picture than to recognize a face. A distinction should also be made between familiar and unfamiliar face images, with the latter used in this study. While familiar face recognition is likely to be robust to changes in image, unfamiliar face recognition is bound more closely to the visual properties of the particular image individuals are viewing [49]. For these reasons, an important question for future research therefore concerns the extent to which the results obtained in this study are robust to changes in image.

Gender, gender distribution of exposed faces, and exposure time are not the only factors that may influence face recognition. Evidence suggests that other personal factors such as age [30]; genetic heritage [50]; prosopagnosia (face blindness) [51, 52]; general motivation for perceiving faces [53]; experience in face recognition [54]; and preferences for processing of different face parts (e.g., eyes, nose, and mouth) [9, 55] may also affect individuals’ face recognition abilities.

The controls imposed by the experimental settings of these studies, though necessary to isolate the effects of exposure time, may arise differently in actual face exposure settings. For example, if face exposures are taking place when the consumer is in a group setting, the potential influence of other people may distort the influence of exposure time on the ability to recognize faces. In the present research the face images were exposed to participants without manipulating the emotional appearance of the exposed faces (e.g., do they look happy, do they look sad, do they look aroused, etc.) and without showing any other parts of the body. Hence, they are many avenues for future research wishing to examine individuals’ face recognition ability in additional and/or other settings than the ones used in the current study.

The context of the importance of facial recognition needs to be considered when relying on prior exposure for insights. Recent developments using Artificial Intelligence (AI) for facial recognition suggest that low-resolution face images are also very hard to recognize and that this is one of the main obstacles of face recognition in surveillance systems [56]. So far AI has not been compared to the accuracy of female versus male human counterparts in various time exposures. This would be an interesting study of comparison, given that the world is investing so much in AI and people are worried about losing their jobs to AI.

## Conclusion

This study examined how the interaction of exposure time with gender and with gender distribution of exposed faces affects an individual’s face recognition performance. Based on a set of four experimental studies several results were obtained. We found that women show a decrease in face recognition ability due to a lower exposure time. However, we also found that when exposure time is short (vs. long) women recognize a larger proportion of same gender faces and also recognize a larger proportion of same gender faces as compared with the proportion of same gender faces recognized by men. Finally, it was shown that when individuals are only exposed to same gender faces, women recognize more faces than men irrespective of exposure time.

## References

[1] Rehnman J, Herlitz A. Women remember more faces than men do. Acta Psychologica. 2007;124(3):344–55. doi: 10.1016/j.actpsy.2006.04.004 16764811

[2] Herlitz A, Lovén J. Sex differences and the own-gender bias in face recognition: A meta-analytic review. Visual Cognition. 2013;21(9–10):1306–36.

[3] Taubert J, Apthorp D, Aagten-Murphy D, Alais D. (2011). The role of holistic processing in face perception: Evidence from the face inversion effect. Vision Research. 2011; 51(11):1273–78. doi: 10.1016/j.visres.2011.04.002 21496463

[4] Laughery KR, Alexander JF, Lane AB. Recognition of Human Faces: Effects of Target Exposure Time, Target Position, Pose Position, and Type of Photograph. Journal of Applied Psychology. 1971;55(5):477–83. 10.1037/h0031646 5113201

[5] Hehman E, Sutherland CAM, Flake JK, Slepian ML. The Unique Contributions of Perceiver and Target Characteristics in Person Perception. Journal of Personality and Social Psychology. 2017;113(4):513–29. doi: 10.1037/pspa0000090 28481616

[6] Sporer SL. Recognizing faces of other ethnic groups: An integration of theories. Psychology. Psychology, Public Policy, and Law. 2001;7(1):36–97.

[7] Verosky SC, Porter J., Martinex JE, Todorov A. Robust Effects of Affective Person Learning on Evaluation of Faces. Journal of Personality and Social Psychology. 2018;114(4):516–28. doi: 10.1037/pspa0000109 29620399

[8] Rule NO, Ivcevic Z, Krendl AC, Ambady N. Accuracy and Consensus in Judgments of Trustworthiness from Faces: Behavioral and Neural Correlates. Journal of Personality & Social Psychology. 2013;104(3):409–26. doi: 10.1037/a0031050 23276271

[9] Wang R, Li J, Fang H, Tian M, Liu J. Individual Differences in Holistic Processing Predict Face Recognition Ability. Psychological Science. 2012;23(2):169–77. doi: 10.1177/0956797611420575 22222218

[10] Russell R, Duchaine B, Nakayama K. Super-recognizers: People with extraordinary face recognition ability. Psychonomic Bulletin & Review. 2009;16(2): 252–57. doi: 10.3758/PBR.16.2.252 19293090 PMC3904192

[11] Palmer MA, Brewer N, Horry R. Understanding gender bias in face recognition: Effects of divided attention at encoding. Acta Psychologica. 2013;142(3): 362–69. doi: 10.1016/j.actpsy.2013.01.009 23422290

[12] Broadbent DE. Perception and Communication. London: Pergamon Press. 1958.

[13] Pashler HE. The Psychology of Attention. Cambridge, MA: MIT Press. 1998.

[14] Sommer W, Hildebrandt A, Kunina-Habenicht O, Schacht A, Wilhelm O. Sex differences in face cognition. Acta Psychologica. 2013;142(1):62–73. doi: 10.1016/j.actpsy.2012.11.001 23232336

[15] Lovén J, Herlitz A, Rehnman J. Women’s own-gender bias in face recognition memory: The role of attention at encoding. Experimental Psychology. 2011;58(4):333–40. doi: 10.1027/1618-3169/a000100 21310695

[16] Hall JK, Hutton SB, Morgan MJ. Sex differences in scanning faces: Does attention to the eyes explain female superiority in facial expression. Cognition and Emotion. 2010;24(4):629–37.

[17] Fiske ST, Taylor SE. Social cognition (2nd ed.). New York: McGraw Hill; 1991.

[18] Tversky A, Kahneman D. Extensional versus intuitive reasoning. The conjunction fallacy in probability judgment. Psychological Review. 1983;90(4):293–315.

[19] Epstein S. The unconscious, the preconscious and the self-concept. In Suls J and Greenwald (Eds.), Psychological perspectives on the self. Hillsdale, NJ: Erlbaum; 1983;2. 219–47.

[20] Guillem F, Mograss M. Gender differences in memory processing: Evidence from event-related potentials to faces. Brain and Cognition. 2005;57(1):84–92. doi: 10.1016/j.bandc.2004.08.026 15629219

[21] McGivern RF, Huston JP, Byrd D, King T, Siegle GJ, Reilly J. (1997). Sex differences in visual recognition memory: Support for a sex-related difference in attention in adults and children. Brain & Cognition. 1997;34(3):323–36. doi: 10.1006/brcg.1997.0872 9292185

[22] Meyers-Levy J, Tybout AM. Schema congruity as a basis for product evaluation. Journal of Consumer Research. 1989;16(1):39–54.

[23] Meyers-Levy J, Maheswaran D. Exploring differences in males and females processing strategies. Journal of Consumer Research. 1991;18(1):63–70.

[24] Jones DC. Social comparison and body image: Attractiveness comparisons to models and peers among adolescent girls and boys. Sex Roles. 2001;45(9–10):645–64.

[25] Palazzo G, Krings F, Hoffrage U. Ethical Blindness. Journal of Business Ethics. 2012;109(3):323–38.

[26] McIntyre AH, Hancock PJB, Frowd CD, Langton SRH. (2016). Holistic Face Processing Can Inhibit Recognition of Facial Composites. Law and Human Behavior. 2016;40(2):128–35. doi: 10.1037/lhb0000160 26436334

[27] Gosselin F, Schyns PG. Bubbles: A technique to reveal the use of information in recognition tasks. Vision Research. 2001;41: 2261"2271. doi: 10.1016/s0042-6989(01)00097-9 11448718

[28] Haig ND. How faces differ: A new comparative technique. Perception. 1985;14: 601"615. doi: 10.1068/p140601 3836392

[29] Haig ND. Investigating face recognition with an image processing computer. In Ellis HD, Jeeves MA, Newcombe F, Young AW. (Eds.), Aspects of face processing. Dordrecht: Martinus Nijhof; 1986:410–25.

[30] Anastasi JS, Rhodes MG. An own-age bias in face recognition for children and older adults. Psychonomic Bulletin & Review. 2005;12(6):1043–47. doi: 10.3758/bf03206441 16615326

[31] Perfect TJ, Harris LJ. Adult age differences in unconscious transference: Source confusion or identity blending? Memory & Cognition. 2003;31(4):570–80. doi: 10.3758/bf03196098 12872873

[32] Hugenberg K, Young SG, Bernstein MJ, Sacco DF. The categorization individuation model: An integrative account of the other-race recognition deficit. Psychological Review. 2010;117(4):1168–87. doi: 10.1037/a0020463 20822290

[33] Lewin C, Herlitz A. Sex differences in face recognition—Women’s faces make the difference. Brain and Cognition. 2002;50(1):121–28. doi: 10.1016/s0278-2626(02)00016-7 12372357

[34] Rehnman J., Herlitz A. Higher face recognition ability in girls: Magnified by own-sex and own-ethnicity bias. Memory. 2006;14(3):289–96. doi: 10.1080/09658210500233581 16574585

[35] Wright DB, Sladden B. An own gender bias and the importance of hair in face recognition. Acta Psychologica. 2003;114(1):101–14. doi: 10.1016/s0001-6918(03)00052-0 12927345

[36] Iyer ES. Unplanned Purchasing: Knowledge of Shopping Environment and Time Pressure. Journal of Retailing. 1989;65(1):40–57.

[37] Bogacz R, Wagenmakers E-J, Forstmann B, Nieuwenhuis S. The neural basis of the speed–accuracy tradeoff. Trends in Neurosciences. 2010;33(1):10–16. doi: 10.1016/j.tins.2009.09.002 19819033

[38] Héroux L, Laroche M, McGown KL. Consumer Product Label Information Processing: An Experiment Involving Time Pressure and Distraction. Journal of Economic Psychology. 1988;9:195–214.

[39] Nisbett RE., Ross L. Human Inference: Strategies and Shortcomings of Social Judgement. Prentice-Hall, Englewood Cliffs, NJ; 1980.

[40] Venkatesh V, Morris MG. Why Don’t Men Ever Stop to Ask for Directions? Gender, Social Influence, and Their Role in Technology Acceptance and Usage Behavior. MIS Quarterly. 2000;24(1):115–39.

[41] Tarr, MJ. Tarrlab Stimuli: The Face-Place Face Database Project: http://face-place.org/

[42] Suri R, Monroe KB. The Effects of Time Constraints on Consumers’ Judgements of Prices and Products. Journal of Consumer Research. 2003;30(June):92–104.

[43] Benson L., Beach LR. The effects of time constraints on the pre-choice screening of decision options. Organizational Behavior and Human Decision Processes. 1996:67: 222–28.

[44] Huber O, Kunz U. Time pressure in risky decision-making: effect on risk defusing. Psychology Science. 2007:49(4): 415–26.

[45] Yang Y, Green SB. Coefficient Alpha: A Reliability Coefficient for the 21st Century? Journal of Psychoeducational Assessment. 2011: 29(4) 377–92.

[46] Bateman CR, Valentine SR. Investigating the Effects of Gender on Consumers’ Moral Philosophies and Ethical Intentions. Journal of Business Ethics. 2010;95(3):393–414.

[47] Serrien DJ, Ivry RB, Swinnen SP. Dynamics of hemispheric specialization and integration in the context of motor control. Nature Reviews Neuroscience. 2006;7(February):160–66. doi: 10.1038/nrn1849 16429125

[48] Burton AM. Why has research in face recognition progressed so slowly? The importance of variability. The Quarterly Journal of Experimental Psychology. 2013:66(8):1467–85. doi: 10.1080/17470218.2013.800125 23742022

[49] Burton AM, Robin SS, Kramer KL, Ritchie RJ. Identity From Variation: Representations of Faces Derived From Multiple Instances, Cognitive Science. 2016; 40:202–23. doi: 10.1111/cogs.12231 25824013

[50] Zhu Q, Song Y, Hu S, Li X, Tian M, Zhen Z, et al. Heritability of the specific cognitive ability of face perception. Current Biology. 2010;20(2):137–42. doi: 10.1016/j.cub.2009.11.067 20060296

[51] Busigny T, Rossion B. Holistic processing impairment can be restricted to faces in acquired prosopagnosia: Evidence from the global/local Navon effect. Journal of Neuropsychology. 2011;5(1):1–14. doi: 10.1348/174866410X500116 21366884

[52] Ramon M, Busigny T, Rossion B. Impaired holistic processing of unfamiliar individual faces in acquired prosopagnosia. Neuropsychologia. 2010;48(4):933–44. doi: 10.1016/j.neuropsychologia.2009.11.014 19944710

[53] Rossion B. The composite face illusion: A whole window into our understanding of holistic face perception. Visual Cognition. 2013;21(2):139–253.

[54] Mondloch CJ, Pathman T, Maurer D, Le Grand R, de Schonen S. The composite face effect in six-year-old children: Evidence of adult-like holistic face processing. Visual Cognition. 2007;15(5):564–77.

[55] Rotshtein P, Geng JJ, Driver J, Dolan RJ. Role of features and second-order spatial relations in face discrimination, face recognition, and individual face skills: Behavioral and functional magnetic resonance imaging data. Journal of Cognitive Neuroscience. 2007;19(9):1435–52. doi: 10.1162/jocn.2007.19.9.1435 17714006 PMC2600425

[56] Haghighat, M, Abdel-Mottaleb, M. Low Resolution Face Recognition in Surveillance Systems Using Discriminant Correlation Analysis. 12th IEEE International Conference on Automatic Face & Gesture Recognition (FG 2017). 2017:912–17.

